# Oligoarray Comparative Genomic Hybridization-Mediated Mapping of Suppressor Mutations Generated in a Deletion-Biased Mutagenesis Screen

**DOI:** 10.1534/g3.112.002238

**Published:** 2012-06-01

**Authors:** Martin R. Jones, Ann M. Rose, David L. Baillie

**Affiliations:** * Department of Medical Genetics, University of British Columbia, Vancouver, British Columbia V6T 1Z4, Canada; † Department of Molecular Biology and Biochemistry, Simon Fraser University, Burnaby, British Columbia V5A 1S6, Canada

**Keywords:** *Caenorhabditis elegans*, oligoarray comparative genomic hybridization, ROL-3, BCC-1, SRAP-1, duplication suppressors

## Abstract

Suppressor screens are an invaluable method for identifying novel genetic interactions between genes in the model organism *Caenorhabditis elegans*. However, traditionally this approach has suffered from the laborious and protracted process of mapping mutations at the molecular level. Using a mutagen known to generate small deletions, coupled with oligoarray comparative genomic hybridization (aCGH), we have identified mutations in two genes that suppress the lethality associated with a mutation of the essential receptor tyrosine kinase *rol-3*. First, we find that deletion of the Bicaudal-C ortholog, *bcc-1*, suppresses *rol-3*–associated lethality. Second, we identify several duplications that also suppress *rol-3*–associated lethality. We establish that overexpression of *srap-1*, a single gene present in these duplications, mediates the suppression. This study demonstrates the suitability of deletion-biased mutagenesis screening in combination with aCGH characterization for the rapid identification of novel suppressor mutations. In addition to detecting small deletions, this approach is suitable for identifying copy number suppressor mutations, a class of suppressor not easily characterized using alternative approaches.

Suppressor screening is an efficient way to generate mutations in developmentally associated genes. In the model organism *Caenorhabditis elegans*, suppressor mutations have given insight into a number of gene functions, facilitating the elucidation of complex developmental mechanisms and pathways (Dorfman *et al.* 2009; Galvin *et al.* 2011; Rohlfing *et al.* 2011; Schumacher *et al.* 2005; Singh and Han 1995). The time and expense required to identify mutations generated in such screens are, however, a major limiting factor in the value of these approaches (Barbazuk *et al.* 1994; Fay and Johnson 2006; Jorgensen and Mango 2002). Alternative strategies to generate and rapidly map suppressor mutations are therefore required.

The advent of high-resolution molecular methods for mapping mutations has, to a certain extent, replaced the need for slower traditional approaches. A number of such methods are available. Principal among these are whole-genome sequencing (WGS) and oligoarray comparative hybridization (aCGH). WGS has been applied to mutant identification in *C. elegans* and shown to be suitable for detecting a variety of DNA lesions (Chu *et al.* 2012; Flibotte *et al.* 2010; Hillier *et al.* 2008; Hobert 2010; Sarin *et al.* 2008; Shen *et al.* 2008), although methods to reliably identify small insertion/deletions (indels) and duplications are still being refined (Smith 2011). Additionally, although WGS is becoming more cost-effective, intensive and specialized bioinformatic analysis is required for mutation identification and validation. However, aCGH analysis, a technique for querying genome alterations to a high resolution (Dhami *et al.* 2005; Selzer *et al.* 2005), is a viable alternative approach that does not require specialized bioinformatic analysis. Several *C. elegans*–specific aCGH platforms have been developed and have been shown to be effective for rapidly identifying novel single-gene deletions (Maydan *et al.* 2007; O’meara *et al.* 2009), as well as large duplications and deficiencies (Jones *et al.* 2007; Lipinski *et al.* 2011; Maydan *et al.* 2010). The use of aCGH circumvents the need for time-consuming genetic mapping methods and is therefore a suitable method for identifying novel mutations generated in suppressor screens.

The creation of genomic lesions suitable for high-resolution mapping with aCGH requires the use of a mutagen capable of creating small deletions. The generation of single-gene deletion mutations (also known as knockout mutations) is routinely performed in *C. elegans* (Barstead and Moerman 2006; Flibotte *et al.* 2010; Gengyo-Ando and Mitani 2000; Moerman and Barstead 2008). UV-TMP is the mutagen of choice for generating knockout mutations as it has been shown to generate a high frequency of deletions with an average detectable size of 10 kb, in addition to a significant number of small indels (Flibotte *et al.* 2010; Gengyo-Ando and Mitani 2000; Kage-Nakadai *et al.* 2012; Liu *et al.* 1999; Yandell *et al.* 1994).

Here we describe the use of a UV-TMP mutagenesis screen for the generation of suppressors of a mutation in the essential receptor tyrosine kinase (RTK), *rol-3*. We then use aCGH to rapidly map the lesions. In all cases, we detect the suppressive lesion, defining two novel loci that suppress *rol-3*–associated lethality.

## MATERIALS AND METHODS

### Strains and genetics

Maintenance and handling of *C. elegans* were performed as previously described (Brenner 1974). Worms were cultured at 20°, unless otherwise stated. All strains are derivatives from Bristol N2 wild-type animals. Strains used were Bristol N2 wild-type, *rol-3(s1040)*, *sDp31(s3067)*, *sDp32(s3068)*, *sDp33(s3069)*, *sDp34(s3071)*, *sDp35(s3074)*, *sDf149(s3072)*, *rol-3(s1040)*; *sEx2693*, *bcc-1(tm3821)*.

### Transgenic arrays

*sEx2693* is a transgenic array comprising the fosmid WRM0262cC02, which contains the genomic region of T06D8.1/*srap-1*.

### UV-TMP mutagenesis and suppressor screening

The suppressor screen strategy was modified from that previously reported (Barbazuk *et al.* 1994). The mutagenesis method used for this *rol-3* suppressor screen was modified from (Flibotte *et al.* 2010). A mixed stage population of BC3129, *rol-3(s1040)*, was harvested at the permissive temperature of 15°, and the worm suspension was incubated in 2 μg/ml TMP in the dark for 1 hr. Worms were then exposed to 90 sec of UV irradiation at 340 microwatts per square centimeter. After treatment, 50 gravid adults were transferred to 10 cm plates seeded with OP50 and cultured at the permissive temperature (15°) for one generation (7–10 days) before being shifted to the restrictive temperature of 20°. Animals were grown for a further 7–10 days. A single animal was isolated from each plate and maintained at the restrictive temperature (20°) to ensure that the suppressor mutation was retained. We estimate that at least 500,000 chromosomes were screened in this analysis.

### aCGH data analysis of UV-TMP suppressors

aCGH was performed using a whole-genome *C. elegans* array designed with overlapping 50-mer probes targeting annotated exons and micro-RNAs (Maydan *et al.* 2007). aCGH sample preparation, hybridization, and analysis were done as previously described (Maydan*et al.* 2007). Copy number aberrations were detected by visual inspection using the SignalMap browser software (Roche Nimblegen Inc., Madison, WI).

### Molecular identification of deficiency breakpoints in *s3071*

PCR amplification across the region of the breakpoint in the strain *rol-3(s1040)*, *sDf149(s3072)* was performed using the appropriate nested primers (available upon request), and purified products were sent for sequencing at Macrogen (Macrogen, Seoul, Korea).

### RNAi analysis

#### Generation of dsRNA:

RNA interference (RNAi) experiments were performed by injection as previously described (Sonnichsen *et al.* 2005).

#### RNAi injection against deletion and duplication suppressor gene candidates:

Annealed dsRNA targeting M7.7, *bcc-1*, T06D8.1, T06D8.5, and F37H8.5 was injected directly into the syncytial gonad of mutant strains to be tested. Injected animals were recovered for 16 hr at 20° in order to lay any eggs present *in utero* prior to injection and were then transferred individually onto fresh NGM agar plates maintained at restrictive temperature (20°). Evidence of suppression or disruption of suppression was assessed after 7–10 days of growth.

### Suppression of *rol-3(s1040)* lethality with transgenic arrays containing *srap-1*

DNA prepared from the fosmid clones WRM0626cC02, WRM0625bF10, and WRM0635dC04 was injected directly into young adult *rol-3(s1040)* animals raised at permissive temperature of 15°. Fosmid DNA was injected at a concentration of 10 ng/μl with 80 ng/μl *dpy-5* carrier DNA. Injected animals were maintained at the restrictive temperature of 20° to select for suppression. Suppression was assayed by screening for viable progeny after 7–10 days of growth.

### Microscopy and image processing

Analysis of mutant and GFP transgenic animals was performed using a ZEISS Stemi SVC11 dissecting microscope with GFP filters and a Zeiss Axioskop with 10×/0.25, 40×/0.65 and 60×/0.85 objective lenses. All pictures were taken using QCapture software (QImaging) with a QImaging digital camera mounted on the Zeiss Axioskop. Images were processed using Photoshop CS4 (Adobe).

## RESULTS AND DISCUSSION

### Suppressors of *rol-3* generated by UV-TMP harbor deletions and duplications that can be detected using aCGH

The fully penetrant, temperature-sensitive lethality of a hypomorphic mutation in *rol-3(s1040)* animals (Johnsen and Baillie 1991) lends itself to the isolation of extragenic suppressors. Previously, eight suppressor mutations defining the two loci *srl-1* and *srl-2* (suppressor of *rol-3* lethality) were generated (Barbazuk *et al.* 1994). Using traditional genetic methods, these suppressors were mapped to chromosomes II and III, respectively. However, further mapping was complicated by the lack of obvious phenotypes in the single mutant animals and a complex *inter se* complementation between specific alleles of *srl-1* and *srl-2* (Barbazuk *et al.* 1994).

Given the relative ease of screening for suppressors of *rol-3*, we undertook to generate *de novo* suppressor mutations that might be suitable for detection by aCGH. Approximately 500,000 UV-TMP–treated genomes were screened, yielding eight suppressors that we have designated *s3067*–*s3074* (Table 1). Although the temperature-dependent larval lethality associated with the *s1040* allele is suppressed in these strains, animals still exhibit the characteristic adult left-handed rolling (LRol) phenotype (data not shown).

**Table 1:** aCGH mapping data for duplications and deletions in the UV-TMP–derived suppressor strains

Designation	Allele	Chr	Left (bp)	Right (bp)	Size (kb)	Type	% Suppression
*sDp31*	*s3067*	II	10054547	15242654	5188	dup	ND
		V	22351	607014	585	dup	ND
*sDp32*	*s3068*	II	10905657	11229193	324	dup	ND
*sDp33*	*s3069*	II	10905657	11229193	324	dup	95.6 (n = 168)
-	*s3070*	ND	ND	ND	ND	ND	ND
*sDp34*	*s3071*	II	10643051	11289830	647	dup	ND
*sDf149*	*s3072*	IV	11087657	11092473	5	del	41.5 (n = 260)
–	*s3073*	ND	ND	ND	ND	ND	ND
*sDp35*	*s3074*	II	11187892	11983055	795	dup	90.1 (n = 177)

bp, base pair coordinate; Chr, chromosome, del, deletion; dup, duplication; ND, not determined.

**Figure 1:**
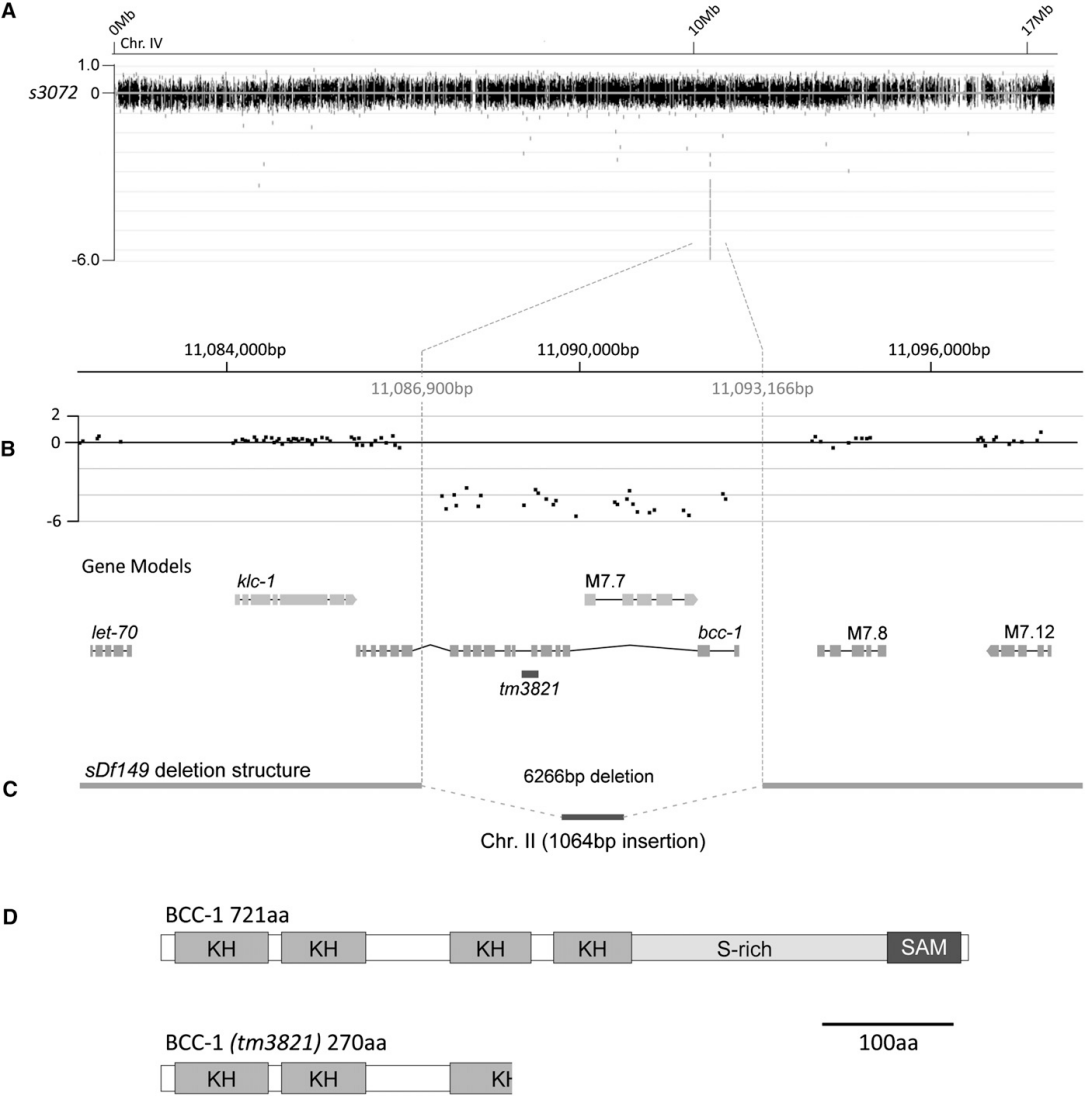
*bcc-1* is a suppressor of *rol-3*. (A) Log2-normalized aCGH data for chromosome IV in the strain *s3072*. (B) An expansion of the region containing a potential deletion. Gene models are shown below the CGH data. (C) Schematic showing the confirmed structure of *sDf149*. The position of the deletion allele *tm3821* is indicated. (D) Protein structure of BCC-1 and the predicted structure of the BCC-1 truncation in *tm3821*. KH, K homology domain; S-rich, serine-rich region; SAM, sterile alpha motif.

We analyzed six of the eight suppressor strains using aCGH. Of these, only *s3072* contains an obvious deletion, located on chromosome IV (Figure 1, A and B). PCR and sequencing of the deleted region in *s3072* animals confirmed the presence of a 6266 bp deletion and 1064 bp insertion from an intergenic region located on chromosome II (Figure 1C). This indel, which we have designated *sDf149*, is a deficiency that disrupts two predicted genes. *sDf149* deletes the complete coding region of the predicted ORF M7.7 and the majority of the gene *bcc-1*, including the ATG start site. M7.7 is an uncharacterized and poorly conserved putative protein kinase. *bcc-1* is an ortholog of the mRNA stabilizing protein Bicaudal-C (Eckmann *et al.* 2002).

### Disruption of *bcc-1* is responsible for suppression of *rol-3* lethality

To determine which of the two genes disrupted by *sDf149* is responsible for the suppression of *rol-3*, we attempted to phenocopy the suppression using RNAi. *rol-3(s1040)* animals are 100% inviable when raised at the restrictive temperature of 20°, arresting at an early larval stage of development (Table 2) (Johnsen and Baillie 1991). To assess suppression by RNAi, *rol-3(s1040)* animals raised at the permissive temperature of 15° were injected with dsRNA targeting either M7.7 or *bcc-1*. These animals were then cultured at the restrictive temperature of 20°, and suppression was assayed by the presence of adults in the progeny of the injected animals. Injection of *rol-3(s1040)* adults with dsRNA targeting M7.7 or a mock injection buffer containing no dsRNA did not produce any viable progeny. Injection of dsRNA targeting *bcc-1*, however, resulted in approximately 10% of the progeny developing to the adult stage (Table 2). This result demonstrates that the suppression is due specifically to disruption of *bcc-1*. To provide further support for this result, we obtained a mutant allele of *bcc-1*, *tm3821*, in which a 517 bp region comprising exon 6 is deleted (Figure 1B). This deletion should lead to truncation of the predicted protein by creating a premature stop codon (Figure 1D). *rol-3(s1040)* animals that are also mutant for *bcc-1(tm3821)* are viable when grown at the restrictive temperature of 20° (41.5%) (Table 1). Together, these data establish that disruption of *bcc-1* alone is sufficient to suppress *rol-3* associated lethality.

**Table 2:** RNAi of suppressor gene candidates in rol-3(s1040) animals maintained at 20°

	RNAi Target			
Phenotype	Mock*^a^*	*bcc-1*	M7.7	Phenotype
Lvl	>500	87	>300	Lvl
Adult	0	8	0	adult
% Suppression	0	9	0	% Suppression

The progeny of 3–10 injected worms was scored.

a A placebo of injection buffer containing no dsRNA was injected as a negative control.

### Five of the suppressors characterized by aCGH contain duplications of chromosome II

**Figure 2:**
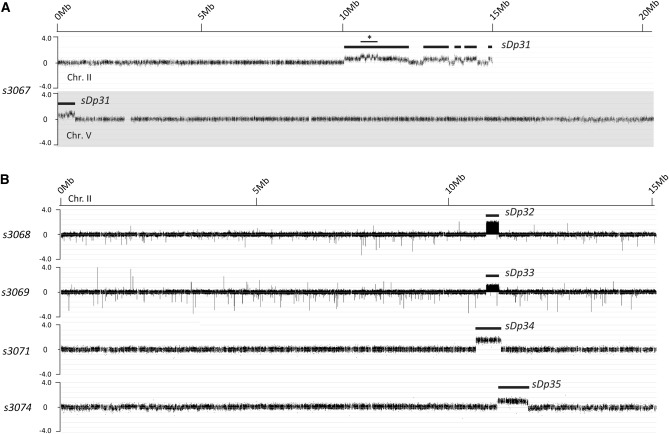
Duplications detected by aCGH in *rol-3(s1040)* suppressor strains. (A) Log2-normalized aCGH data for chromosomes II and V in the strain *s3069*. This strain contains a complex arrangement of duplications covering Chr. II and a single 0.6 Mb duplication of Chr. V. Solid lines above the array data highlight the extent of the duplicated regions. A region containing potential multiple duplications is indicated (asterisk). (B) Log2-normalized aCGH data for chromosome II in the remaining four suppressor strains found to contain duplications.

The genomes of the five remaining suppressors analyzed with aCGH (*s3067*, *s3068*, *s3069*, *s3071*, and *s3074*) do not contain any obvious deletions. However, all genomes contain relatively large duplications of chromosome II. We have designated these duplications *sDp31*–*sDp35* (Figure 2 and Table 1). In addition to several duplicated regions of chromosome II, the suppressor strain *s3067* contains a duplication of 0.6 Mb of DNA from the left end of chromosome V (Figure 2A and Table 1). The duplications *sDp32* and *sDp33* appear to be identical in size; however, based on the probe data, the duplication is present at a higher copy number in *sDp32* (Figure 2B).

### Duplications of the predicted mucin T06D8.1 suppress *rol-3* lethality

**Figure 3:**
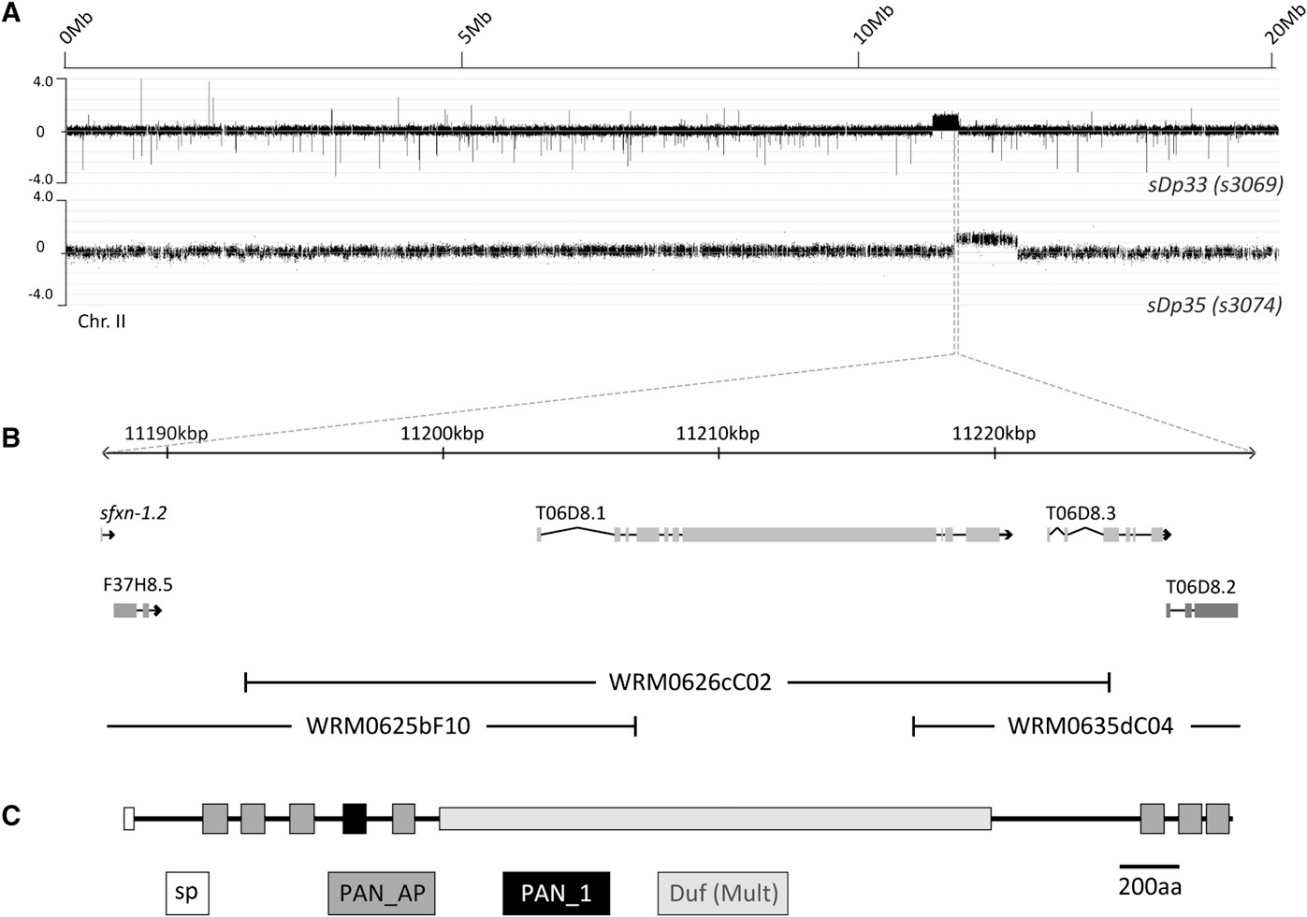
Overexpression of the predicted mucin SRAP-1 (T06D8.1) suppresses *rol-3* lethality. (A) A schematic showing the region common to all duplication-containing suppressor strains. (B) Fosmids used for transgenic suppression experiments are shown below the gene models. (C) The predicted structure of SRAP-1. Duf (Mult), multiple copies of a domain of unknown function; PAN_AP, PAN Apple domain; PAN_1, PAN1 domain; sp, signal sequence.

The prevalence of duplications in the suppressed animals suggested that their insertion into the genome might have disrupted the function of a specific gene, giving rise to the suppression. Alternatively, extra copies of a gene, or genes, present in the duplicated regions might be responsible for the suppression. That the five duplications identified by aCGH are not randomly distributed across the genome but overlap the same region of chromosome II suggested that the latter hypothesis was more likely. The genomic region common to the duplications is around 40 kb in size and contains only three complete ORFs: T06D8.1, a predicted mucin; T06D8.3, a predicted lipid phosphate phosphatase of the PAP2 family; and F37H8.5, the *C. elegans* homolog of gamma-interferon–inducible lysosomal thiol reductase (Figure 3A).

To determine whether one of the three duplicated genes is responsible for the suppression, we attempted to increase the copy number of individual candidate genes by introducing gene-specific fosmids as transgenic arrays (Figure 3B). We injected DNA directly into *rol-3(s1040)* animals raised in the permissive temperature of 15° and screened the progeny of these animals for viability at the restrictive temperature of 20°. Using this approach, we found that only a fosmid that encompasses the complete coding region of T06D8.1 (WRM0626cC02) is capable of rescuing *rol-3*–associated lethality (*sEx2693*). Transgenic arrays composed of fosmids containing either of the adjacent genes, T06D8.3 or F37H8.5, were unable to confer suppression (Figure 3B and Table 3).

**Table 3:** Fosmids used for transgenic suppression of rol-3(s1040) animals maintained at 20°

Fosmid	Genomic Region	Gene Target	*rol-3(s1040)* Viability at 20°
WRM0613dF11	II: 11190820–11222798	T06D8.1	Yes
WRM0625bF10	II: 11169946–11207249	F37H8.5	No
WRM0635dC04	II: 11217009–11252193	T06D8.3	No

It is likely that overexpression of T06D8.1 mediates the suppression of *rol-3*. To provide further support for this finding, we surmised that knockdown of T06D8.1 by RNAi in suppressed *rol-3* mutant strains would abrogate suppression, leading to targeted lethality. Injection of dsRNA targeting T06D8.1 in wild-type animals does not result in lethality (Table 4. However, RNAi targeting T06D8.1 in suppressor strains containing the duplications *sDp33* and *sDp35* completely abolishes suppression (Table 4). This effect is specific to T06D8.1, because introduction of dsRNA targeting the two other candidate genes, T06D8.3 and F37H8.5, does not disrupt the suppression. Together, these data demonstrate that suppression of *rol-3* in the duplication-containing strains is due specifically to the presence of extra copies of T06D8.1. Furthermore, abrogation of the suppression by RNAi knockdown reveals that the suppression is due to overexpression of T06D8.1 (Table 4). T06D8.1 encodes a predicted mucin similar to the serine-rich adhesion molecule SraP from *Staphylococcus aureus* (McKay *et al.* 2003). We have renamed this gene *srap-1* (for serine rich adhesion protein-like).

**Table 4:** RNAi against suppressor candidates in duplication suppressed animals maintained at 20°

	% Viability of Strains Targeted by RNAi			
Strain	Mock*^a^*	T06D8.1	T06D8.3	F37H8.5
Bristol N2 (Wild type)	100	100	100	100
*rol-3(s1040)*; *sDp33*	90	0	80	97
*rol-3(s1040)*; *sDp35*	95	0	95	95

The progeny of 3–10 injected worms was scored.

a A placebo of injection buffer containing no dsRNA was injected as a negative control.

## CONCLUSIONS

This study describes a straightforward approach for the rapid identification of *de novo* suppressor mutations. Using a deletion-biased mutagenesis screen combined with high-resolution aCGH mapping, we have identified two novel loci that suppress the lethality associated with a temperature-sensitive mutation of the essential RTK *rol-3*. These two loci represent the first suppressors of this gene to be identified at the molecular level. The approach we have described does not require complex sample preparation or specialized informatics analysis beyond the scope of standard laboratory techniques. Additionally, and perhaps most significantly, this approach can be used to rapidly characterize copy number suppressors to a high resolution. This is something that is not easily achieved using alternative approaches, such as WGS. The use of aCGH opens up the possibility of tailoring suppressor screens for the isolation of dominant suppressors that can be quickly assayed for the presence of duplications. Investigations of this type will facilitate a better understanding of the consequences of altering expression levels of genes that function in important biological pathways.
